# Beyond GCP: developing a differentiated competency framework for principal investigators in innovative drug clinical trials in China

**DOI:** 10.3389/fmed.2026.1933721

**Published:** 2026-09-23

**Authors:** Bo Sun, Jiling Li, Ying Xu, Leilei Zhu, Gang Li, Jing Zhang, Zhenglong Dong, Meicheng Yang, Xiangyu Li

**Affiliations:** 1 Shanghai Center for Drug Evaluation and Inspection, Shanghai, China; 2 Shanghai Pulmonary Hospital, School of Medicine, Tongji University, Shanghai, China; 3 Clinical Research Center, Shuguang Hospital Affiliated to Shanghai University of Traditional Chinese Medicine, Shanghai, China; 4 Clinical Pharmacology Research Center, Huashan Hospital, Fudan University, Shanghai, China

**Keywords:** clinical trial, Delphi method, differentiated competency framework, innovative drug, principal investigator

## Abstract

**Background:**

The increasing complexity and diversity of innovative drug clinical trials in China have expanded the competencies expected of principal investigators (PIs) beyond minimum regulatory qualifications. However, existing frameworks are primarily international and may not fully reflect China's institutional and regulatory context. This study aimed to develop a concise PI competency inventory adapted to China and to explore a differentiated, context-sensitive approach to competency development.

**Methods:**

A multiphase mixed-methods modified Delphi study was conducted. First, an initial pool of 64 competency indicators was generated through a structured literature review and semi-structured expert interviews. Subsequently, two rounds of the Delphi method were conducted. In the initial round, a comprehensive expert recruitment process was undertaken, leading to indicator integration through quantitative correlation and content analyses which streamlined the indicators to 30 items. The second-round consultation was conducted using the streamlined indicators.Panel composition changed between rounds: some Round 1 experts did not participate in Round 2, and two regulatory experts were added to strengthen stakeholder representation and content validity. The Statistical Package for the Social Sciences (SPSS) 26.0 was utilized for quantitative analysis, encompassing descriptive statistics, the calculation of Kendall's *W* and the coefficient of authority (Cr).

**Results:**

An exploratory framework comprising 30 competencies was established. The mean expert authority coefficient was 0.87. The test of concordance was statistically significant (*p* < 0.001), but Kendall's *W* was 0.206, indicating limited inter-expert concordance. Indicators were categorized into three descriptive strata by coefficient of variation (CV): high-consensus (CV ≤ 7%; 4 items), medium-consensus (7% < CV ≤ 17%; 19 items), and low-consensus (high-divergence) (17% < CV < 25%; 7 items). These strata describe variability in the panel's ratings and do not establish differences in competency importance or a developmental sequence.

**Conclusions:**

This exploratory 30-item inventory provides an initial structure for evaluating PI competencies in China's innovative-drug trial context. Its differentiation logic offers preliminary perspectives to inform the future development of differentiated and context-sensitive PI competency models. While this framework may inform future training system design, institutional human-resource management, and PI career development, its long-term validity, feasibility, and practical utility warrant prospective, real-world evaluation.

## 1 Introduction

The global wave of pharmaceutical innovation, together with China's strategic transformation from generic-drug development toward original innovation, has increased the quantity and complexity of clinical trials for innovative drugs in China (1, 2). According to the Annual Report on the Progress of New Drug Registration Clinical Trials in China, a total of 5,215 clinical trials were registered on the Drug Clinical Trial Registration and Information Publicity Platform in 2025 (3). Within this ecosystem, the role of the principal investigator (PI) has undergone a fundamental transformation. Rather than implementing study protocols or serving as attending physicians for trial participants, they have increasingly become hubs for integrating scientific research, management, ethics, commercial value, and collaborative responsibilities (4). Emerging trial paradigms, including cell and gene therapy, personalized tumor immunotherapy, decentralized clinical trials, artificial intelligence and real-world data, may require PIs to possess interdisciplinary insight, the ability to integrate and apply new technologies, and an in-depth understanding of the global research and development landscape (5–7).

However, frameworks for evaluating PI competency remain incompletely adapted to the Chinese innovative-drug trial context. ICH GCP and its national derivatives define investigator responsibilities but do not constitute operational competency-assessment instruments (8–12). Existing frameworks address complementary purposes. The Joint Task Force for Clinical Trial Competency (JTF) framework provides a comprehensive, level-based model for the broader clinical research workforce. The TDR framework supports the development of competency across entire research teams and diverse settings. The ACRP guidelines translate PI responsibilities into practical expectations for training, job descriptions, oversight, and delegation (13–22).

The relevance of a competency framework also depends on the regulatory and institutional context in which PIs work. Accordingly, differences in PI governance between China and the United States should be considered when adapting international frameworks. The United States implements a clear direct-responsibility mechanism for clinical trials, explicitly designating PIs as the primary site-level responsible entities. PIs bear personal, non-delegable responsibility for the compliance of trial implementation, the authenticity of data and the safety of trial participants, all of which serve as core inspection targets during FDA inspections. Severe regulatory non-compliance or research misconduct may result in formal disqualification of the investigator. This system grants PIs substantial operational autonomy while holding them strictly accountable as individuals (23, 24). China implements a dual-subject accountability system involving both institutions and PIs. Clinical trials must be conducted through registered institutions, and PIs are required to complete filing procedures within the institution; regulatory penalties are generally imposed on both institutions and PIs (25, 26). At the statutory and regulatory level, clear qualification requirements for PIs in drug clinical trials are specified in the Regulations for the Administration of Drug Clinical Trial Institutions (27) and the Key Points and Judgment Principles for Supervision and Inspection of Drug Clinical Trial Institutions (Trial) (28), namely that they shall hold a senior professional title and have participated in more than three drug clinical trials. These differences have led to distinctions between Chinese and American PIs in terms of responsibility boundaries, behavioral incentives, risk perception and competence development pathways. Simply transplanting existing international competence models without considering the local context would make it difficult to accurately evaluate the competence of PIs in China.

At present, the ambiguity and homogeneity in PI competency evaluation have led to multiple practical dilemmas. For senior PIs, repeated assessment of basic compliance competency fails to identify their high-level strategic value. For novice PIs, the cumbersome list of competencies makes it difficult for them to formulate a clear growth path. For sponsors and research institutions, there is a lack of scientific basis for decision-making when selecting PIs for specific types of trials. For the entire industry, the allocation of training resources may deviate from actual competency gaps, affecting input-output efficiency.

Therefore, there is a lack of a China-adapted, differentiated PI competency indicator system to support the high-quality development of innovative drug clinical trials in China. This study aims to address this demand and achieve the following research objectives through a sequential explanatory mixed-methods design: (1) to establish a streamlined and comprehensive core competency indicator system for PIs adapted to China's regulatory and institutional context; (2) to quantitatively analyze the degree of consensus and dispersion for each competency; (3) to propose an exploratory and differentiated PI competency development framework based on the results of the consensus analysis, thereby offering preliminary theoretical perspectives that can inform the professional development of PIs, the refined management of institutions, and the progress of the industry.

## 2 Methods

### 2.1 Study design

A multiphase mixed-methods modified Delphi design was adopted in this study (29, 30). The research was conducted across three linked phases: initial indicator item pool development through a structured literature review and semi-structured expert interviews; quantitative refinement and scoring via two Delphi rounds; and qualitative interpretation of expert comments integrated with the quantitative findings. Panel composition changed between rounds: 29 of the 50 valid Round 1 respondents participated in Round 2, and two additional regulatory experts were recruited to improve stakeholder balance and content validity, yielding a reconstituted 31-member panel (Figure 1).

**Figure 1:**
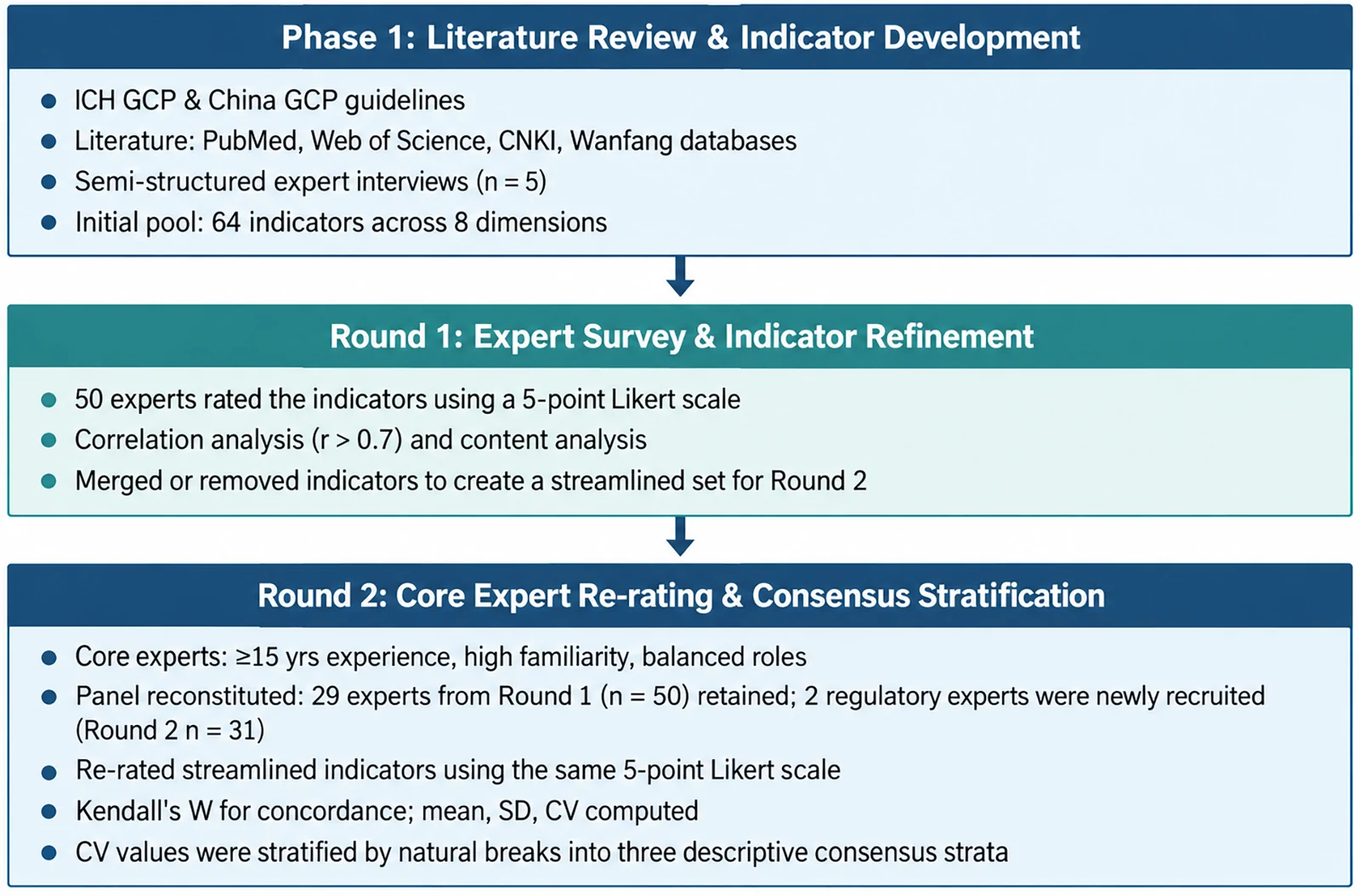
Overview of the Delphi process. CV, coefficient of variation; GCP, good clinical practice; ICH, International Council for Harmonisation; PI, principal investigator; SD, standard deviation.

#### 2.1.1 Construction of the initial indicator pool

This study first conducted a structured literature search across databases including PubMed, Web of Science, China National Knowledge Infrastructure (CNKI), and Wanfang using keywords related to “investigator competency” (14–18, 20, 21). Meanwhile, relevant requirements in ICH GCP (8) and China GCP (9) were reviewed. These sources were combined with semi-structured expert interviews (*n* = 5) to develop a preliminary pool of 64 competency items (Supplementary Table 1). These items span eight theoretical dimensions: scientific mindset and study design, ethics and trial participant protection, innovative drug R&D and regulatory compliance, clinical research operations management, research site and project management, data management and quality control, leadership and professional literacy, and communication and collaboration.

#### 2.1.2 Indicator optimization and consensus formation

Two rounds of the Delphi method were conducted. The first round of the Delphi method aimed to streamline the indicators in the initial pool and select experts for the second round. Quantitative statistical methods (correlation analysis) and content analysis were subsequently used to optimize the indicators to form a streamlined indicator system, and the second round of the Delphi method was then used to administer structured questionnaires and analyze the revised indicator system (31–33).

#### 2.1.3 In-depth interpretation of results

Based on the quantitative results of the second-round consultation (especially for indicators with high dispersion) and the expert comments and feedback collected during the Delphi process, in-depth analyses and interpretations were conducted to provide potential interpretive perspectives.

## 3 Delphi method

### 3.1 First round of Delphi method

This Delphi round aimed to evaluate the 64 candidate competency indicators to identify and merge highly correlated redundant indicators and eliminate relatively unimportant items. It also provided information used to invite a smaller, highly engaged group for the second Delphi round.

#### 3.1.1 Expert selection

Purposive sampling was used to recruit experts nationwide, with most participants based in Shanghai. Eligibility criteria were: (a) at least 5 years of experience in drug clinical trial-related work; (b) relevant senior experience in four fields: clinical trial institution management, regulatory science, PI practice in clinical research, or sponsor R&D; (c) an associate senior professional title or an equivalent position; and (d) willingness to participate in multiple rounds of consultation.

#### 3.1.2 Tool

An electronic questionnaire containing 64 initial indicators was distributed. Experts rated the importance of each indicator on a 5-point Likert scale (1 = extremely unimportant, 5 = extremely important), and could provide written comments.

#### 3.1.3 Indicator optimization

A combined strategy of correlation analysis and content analysis was adopted for indicator optimization in this study (34). The Pearson correlation coefficient matrix among the 64 initial indicators was calculated. According to relevant standards (35), indicator pairs with a correlation coefficient greater than 0.7 can be identified as highly correlated, which prompted review for potential redundancy, but it did not trigger automatic merging. The research team conducted content review and discussion of highly correlated indicator pairs, and merged them after confirming their wording and conceptual overlap and considering expert feedback. Meanwhile, indicators with relatively lower mean importance scores were carefully eliminated with reference to expert comments.

### 3.2 Second round of Delphi method

The second round of the Delphi method was conducted using the indicators streamlined in the first round. Experts rated importance scores for each indicator, and the resulting data were used to estimate overall concordance and describe dispersion.

#### 3.2.1 Expert selection

A core expert group was selected from the first-round experts based on the following criteria: (a) more than 15 years of work experience in clinical trial-related fields; (b) self-evaluated familiarity with the topic as “very familiar” or “relatively familiar” in the first round; (c) engagement was operationally defined by the questionnaire completion time, with those exceeding the mean duration considered as providing more deliberate responses in the first round; (d) the number of experts in each role was deliberately balanced to ensure the framework would reflect the perspectives of multiple stakeholders. Completion time was used only as a pragmatic indicator of engagement, not as a validated measure of response quality. The two additional regulatory experts were invited to improve stakeholder balance. These choices produced a reconstituted panel for the second round. This purposive sampling approach was intentionally adopted to safeguard the content validity of the framework. By ensuring that regulatory perspectives were sufficiently represented, the resulting indicator system better reflects the multidimensional requirements of the clinical trial ecosystem, rather than being skewed by a single stakeholder group.

#### 3.2.2 Tool

An electronic questionnaire containing the streamlined indicators was distributed. Experts rated the importance of each indicator using the same 5-point Likert scale and could provide written comments.

## 4 Data analysis strategy

Statistical analysis was performed using SPSS 26.0 software.

### 4.1 The activeness and authority of experts in the Delphi method

The valid response rate of questionnaires was used to measure expert activeness. The expert authority coefficient (Cr), used to evaluate professional credibility, was calculated as the arithmetic mean of the judgment coefficient (Ca) and familiarity coefficient (Cs) (34). A Cr value ≥0.7 was considered acceptable.

### 4.2 Concordance Analysis

Kendall's coefficient of concordance (*W*) was used to assess agreement among experts. A higher Kendall's *W* indicates greater agreement among the experts (0 ≤ *W* ≤ 1), and *p* < 0.05 is considered statistically significant (36).

### 4.3 Descriptive statistics

The mean (M), standard deviation (SD), and coefficient of variation (CV = SD/M) of each indicator were calculated. CV was used as a relative index to reflect the dispersion of expert opinions. Items with CV < 0.25 were defined as having reached expert consensus (37). In this study, indicators with a CV ≥ 0.25 were considered for exclusion.

In the second round of the Delphi method, this study further adopted a data-driven stratification strategy to stratify CV values into intervals to finely distinguish differences in the level of expert consensus across various PI competency indicators. The specific procedures were as follows: (a) sorting the CV values of all indicators in ascending order and observing their natural distribution characteristics; (b) in light of the actual distribution pattern of CV values, drawing on the principle of cluster analysis that “indicators with similar statistical characteristics are categorized into the same group”, and dividing intervals based on natural breaks after sorting (38); and (c) integrating data distribution characteristics and indicator connotations and classifying all PI competency indicators into three descriptive consensus strata: high-consensus, medium-consensus and high-divergence (low-consensus).

## 5 Results

### 5.1 Composition of the expert panel

A total of 55 experts were recruited in the first round of the Delphi method. 50 returned valid responses, and 29 of these respondents participated in the second round. Two additional regulatory experts were recruited to improve stakeholder balance and content validity (Figure 2).

**Figure 2:**
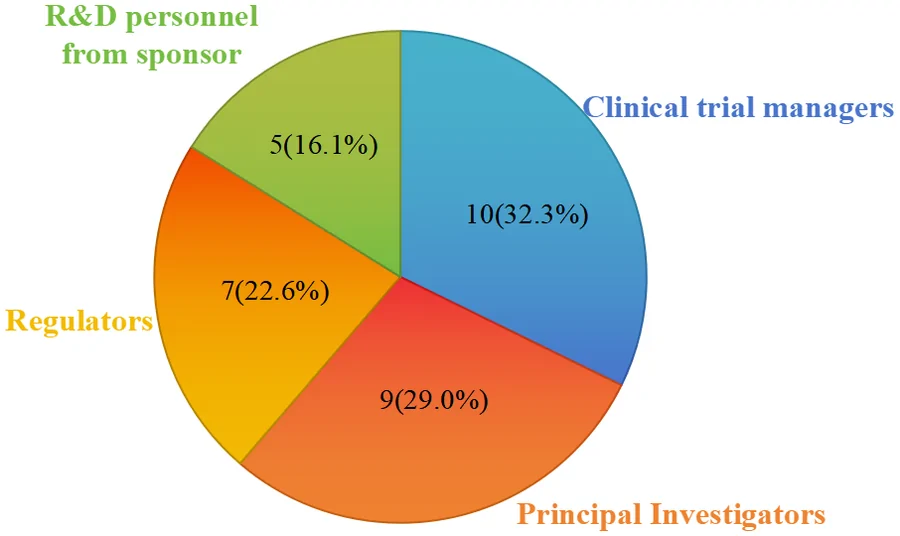
Composition of the experts (the 2^nd^ round)*panel reconstituted: 29 of the original 50 round 1 experts retained, with 2 newly recruited regulatory experts.

The final expert panel of the second round comprised 31 participants, categorized into four stakeholder groups to ensure comprehensive representation across the clinical trial ecosystem. Clinical-trial institution managers (*n* = 10, 32.3%) were primarily recruited from the management offices of major clinical-trial institutions. Eight of them were qualified national drug inspectors, and seven had experience as PIs, thereby offering integrated perspectives on institutional administration, regulatory oversight, and front-line research practice. All regulators (*n* = 7, 22.6%) were from national or provincial drug evaluation and inspection departments with national inspector qualifications, representing regulatory requirements and compliance benchmarks. Principal investigators (*n* = 9, 29.0%) were clinicians engaged in innovative drug clinical trials; two also held inspector qualifications. This group provided clinical practice perspectives. R&D personnel from sponsors (*n* = 5, 16.1%) worked in clinical R&D departments of domestic or international pharmaceutical companies, representing sponsors' expectations regarding PI competency. The composition provided perspectives from regulatory practice, institutional management, clinical practice, and sponsor development.

### 5.2 Expert activeness and authority

In the first round of the Delphi method, 55 questionnaires were distributed and 50 valid responses were returned, yielding a valid response rate of 90.9%. A total of 31 experts were finally enrolled for the second round, with a valid response rate of 100%, indicating a high level of expert activeness.

The mean expert authority coefficient (Cr) in the second round was 0.87 (range: 0.65–0.95, SD = 0.086), which exceeded the acceptable threshold of 0.70. This confirmed that the recruited expert group possessed high professional authority, thus ensuring the credibility of the research findings.

### 5.3 Results of the first round of Delphi method and indicator refinement

Quantitative correlation analysis and content analysis were conducted on the data from the first round to optimize the initial pool of 64 competency indicators. For instance, the original indicators “Analyze mechanisms based on pathophysiology and pharmacology” and “Formulate monitoring indicators using toxicological data” were highly correlated and both fell under the domain of mechanism understanding and safety design, and were therefore merged into one integrated competency (Indicator 1). Scattered items concerning GCP compliance, personal information protection, protection of vulnerable populations, dynamic risk–benefit assessment, and SAE reporting were consolidated into a comprehensive and logically coherent ethical compliance competency (Indicator 11). The original indicator “Proficient in EDC system operation and standardized data entry and storage,” which had a high coefficient of variation (CV = 30.85%), was removed. Ultimately, the initial indicator pool was systematically integrated and refined into a list of 30 core competencies (Table 1 and Supplementary Table 2), which was adopted as the evaluation tool for the second Delphi round.

**Table 1:** Descriptive statistics for the 30 optimized competency indicators after two Delphi rounds.

No.	Optimized indicators	Min	Max	Mean	SD	CV
1	Integrate pathophysiology, pharmacology, and toxicology to analyze the mechanism of action of innovative drugs, guide intervention design, and formulate safety monitoring indicators.	3	5	4.58	0.720	15.72%
2	Based on epidemiological characteristics of diseases in China, formulate research hypotheses that meet clinical needs; identify testable scientific questions and translate them into research hypotheses.	2	5	4.55	0.723	15.89%
3	Define efficacy/safety endpoints in compliance with NMPA review requirements.	3	5	4.81	0.543	11.29%
4	Lead the evaluation and optimization of screening criteria and implementation procedures in multicenter clinical trial protocols to ensure consistency and high-quality research execution across regions in China.	4	5	4.77	0.425	8.91%
5	Evaluate and optimize trial protocols to ensure scientific rigor and ethical appropriateness, balancing quality with participant risk.	4	5	4.84	0.374	7.73%
6	Integrate innovative technologies (e.g., novel biomarkers, digital tools) into innovative drug research to drive data-informed continuous improvement of research implementation and decision-making.	3	5	4.13	0.718	17.38%
7	Critically analyze clinical trial data to identify efficacy and safety signals, assessing the application potential and limitations of research findings.	3	5	4.48	0.677	15.11%
8	Propose pathways for translating research findings into clinical practice in China.	3	5	4.39	0.761	17.33%
9	Explain the exploratory nature of innovative drug research to participants and avoid therapeutic misconception (i.e., understanding the principle of clinical equipoise).	3	5	4.61	0.615	13.34%
10	Design protocols that clearly distinguish between routine clinical care and research procedures.	3	5	4.55	0.568	12.48%
11	Comply with Chinese GCP, ICH GCP, the Declaration of Helsinki, and the Personal Information Protection Law; attend to safety event reporting requirements; dynamically assess participant risk–benefit ratio; and provide care for vulnerable populations.	4	5	4.90	0.301	6.14%
12	Understand registration requirements of major global regulatory authorities (including China) and incorporate them into trial design and execution.	2	5	4.23	0.884	20.90%
13	Cooperate with sponsors to complete data collection and documentation at all stages, meeting Chinese regulatory submission requirements, and understand the full lifecycle of innovative drug R&D.	2	5	4.42	0.848	19.19%
14	Lead compliance with Chinese GCP and research center SOPs.	4	5	4.87	0.341	7.00%
15	Supervise team members’ compliance to ensure all research activities strictly follow the protocol, preventing data fabrication and procedural irregularities.	4	5	4.90	0.301	6.14%
16	Execute procedures for receipt, storage, dispensing, return, and destruction of investigational products (including cold chain management), with accurate record-keeping to ensure account–inventory consistency.	2	5	4.26	0.999	23.45%
17	Regularly report research progress to sponsors and IRB/IEC.	2	5	4.39	0.761	17.33%
18	Ensure source data (medical records, laboratory reports, etc.) are authentic, accurate, and complete.	2	5	4.84	0.583	12.05%
19	Participate in evaluating the Data Management Plan (DMP) and Statistical Analysis Plan (SAP) to ensure that research objectives are achieved scientifically and efficiently.	3	5	4.19	0.749	17.88%
20	Cooperate in database locking to ensure data readiness for statistical analysis.	2	5	4.58	0.720	15.72%
21	Cooperate with external monitoring and auditing; establish internal QC procedures; rectify quality issues for continuous improvement.	3	5	4.68	0.541	11.56%
22	Establish a team training and development system with clear responsibilities, enhancing overall professional competence through mentoring and performance management.	4	5	4.71	0.461	9.79%
23	Prevent academic misconduct (e.g., data fabrication, non-compliant informed consent).	4	5	4.90	0.301	6.14%
24	Proactively declare research-related conflicts of interest in compliance with research integrity requirements.	4	5	4.74	0.445	9.39%
25	Continuously monitor policies and technological advances for innovative drugs in China, and integrate new policies and technologies into research practice.	3	5	4.42	0.620	14.03%
26	Communicate efficiently with project teams (CRC, CRA, laboratory) to ensure smooth processes; maintain timely communication with IRB/IEC and NMPA to respond to regulatory requirements.	3	5	4.81	0.477	9.92%
27	Collaboratively resolve research issues (e.g., protocol deviations, SAE management).	4	5	4.81	0.402	8.36%
28	Provide feedback on practical issues to offer decision-making references for sponsors.	3	5	4.45	0.568	12.76%
29	Lead or serve as a major contributor in writing high-quality academic papers and publishing research findings in peer-reviewed journals.	3	5	4.39	0.715	16.29%
30	Educate the public on the value of innovative drug research to enhance social awareness.	3	5	4.32	0.653	15.12%

Mean, standard deviation (SD), and coefficient of variation (CV = SD/Mean) are computed from Round 2 importance ratings on a 5-point Likert scale (1 = extremely unimportant, 5 = extremely important). Across the 30 optimized indicators, Round 2 mean importance ratings ranged from 4.13 to 4.90 (mean = 4.58), and CV values ranged from 0.061 to 0.235. Indicators are classified into high-, medium-, and low-consensus strata on the basis of CV.

### 5.4 Results of the second round of Delphi method

#### 5.4.1 Overall concordance and item-level dispersion

The mean importance score exceeded 4.0 for all 30 indicators, indicating that the experts generally considered the indicators important. The Kendall's *W* was 0.206 with a *p*-value of less than 0.001. Based on the *p*-value, the null hypothesis of no concordance was rejected. However, the relatively low value of Kendall's *W* indicates that the experts had only limited agreement regarding the relative rankings of the indicators. Therefore, the statistical significance of the concordance test should not be interpreted as evidence of strong expert consensus.

#### 5.4.2 CV-based rating dispersion across PI competency indicators

Across these 30 indicators, Round 2 importance ratings ranged from 4.13 to 4.90 on the 5-point scale, with an overall mean of 4.58 (SD = 0.58) (Supplementary Table 3). Coefficients of variation ranged from 0.061 to 0.235. Using the predefined CV classification procedure, four indicators (13.3%) were assigned to the high-consensus stratum, 19 (63.3%) to the medium-consensus stratum, and seven (23.3%) to the low-consensus (high-divergence) stratum (Table 2). Complete item-level results are provided in Supplementary Table 3.

**Table 2:** Classification of the 30 PI competency indicators into three descriptive consensus Strata.

Descriptive consensus stratum	High-consensus	Medium-consensus	Low-consensus (high-divergence)
*n* (%)	4 (13.3%)	19 (63.3%)	7 (23.3%)
Mean importance score (5-point scale)	4.87–4.90	4.32–4.84	4.13–4.42
Coefficient of Variation (CV)	6.14%–7.00%	7.73%–16.29%	17.33%–23.45%

Full Round 2 statistics (Min, Max, Mean, SD, CV) and hierarchical cluster membership for all 30 indicators are provided in Supplementary Table 3.

To explore whether the retained indicators formed distinguishable rating profiles, we performed a hierarchical cluster analysis of the Round 2 ratings. The analysis yielded a three-cluster solution (Cluster 1, *n* = 4; Cluster 2, *n* = 25; Cluster 3, *n* = 1; Supplementary Table 3). The three clusters did not map one-to-one onto the CV-based consensus strata, suggesting that multivariate rating patterns captured aspects of panel agreement not fully reflected by CV alone. Given that two clusters contained only four indicators and one indicator, respectively, these groupings should be interpreted as exploratory and will require confirmation in larger independent panels.

## 6 Discussion

As summarized in Supplementary Table 4, existing competency frameworks are internationally focused and not intended as a compact assessment instrument for PIs engaged in innovative drug trials under China's regulatory and institutional framework. In this context, the present study does not seek to replace them but rather to provide a preliminary, exploratory 30-item inventory, one that incorporates the viewpoints of regulatory authorities, clinical trial institutions, investigators, and sponsors, and that is adapted to the Chinese context while easing the burden of routine application. It should be emphasized, however, that both the validity and the practical value of this inventory await future prospective evaluation.

In China, although the qualification and supervision of PIs for clinical trials fall under the drug regulatory authority, PIs are physicians whose primary professional practice is governed by the health-care system. Current appraisal emphasizes clinical and scientific credentials rather than systematic competency in conducting clinical trials. Although international frameworks such as the JTF are comprehensive, their routine application can be complex and time-consuming, and expert feedback indicated that the initial 64-item set would be difficult to implement in routine practice. To address this implementation gap, we reduced the inventory to 30 items and integrated the perspectives of drug regulators, clinical trial institutions, investigators, and sponsors, to propose an exploratory PI competency framework featuring focused priorities and enhanced practical feasibility for reference.

### 6.1 Exploratory interpretation of rating dispersion in PI competency indicators

The three CV-based strata provide a descriptive summary of relative rating dispersion within the Round 2 panel, and their substantive interpretation remains exploratory (Figure 3). Lower dispersion among indicators related to ethical compliance and research integrity may reflect comparatively stable agreement within the panel. Intermediate dispersion was observed primarily among operational indicators, whereas higher dispersion characterized indicators related to global regulatory requirements, emerging technologies, and clinical translation, which largely represented context-sensitive capabilities. Indicators in both the intermediate- and higher-dispersion strata nevertheless received high importance ratings. The intermediate level of dispersion indicates moderate but non-negligible variability across the panel without implying a lack of overall endorsement. In the higher-dispersion stratum, the greater variability, interpreted alongside indicator content and expert comments, may reflect differences in professional roles, institutional resources, regulatory environments, and practice contexts rather than lower importance or insufficient endorsement. The vertical arrangement in Figure 3 is intended solely to visualize relative rating dispersion and should not be interpreted as indicating differences in competency importance, validated competency levels, or an inherent developmental hierarchy.

**Figure 3:**
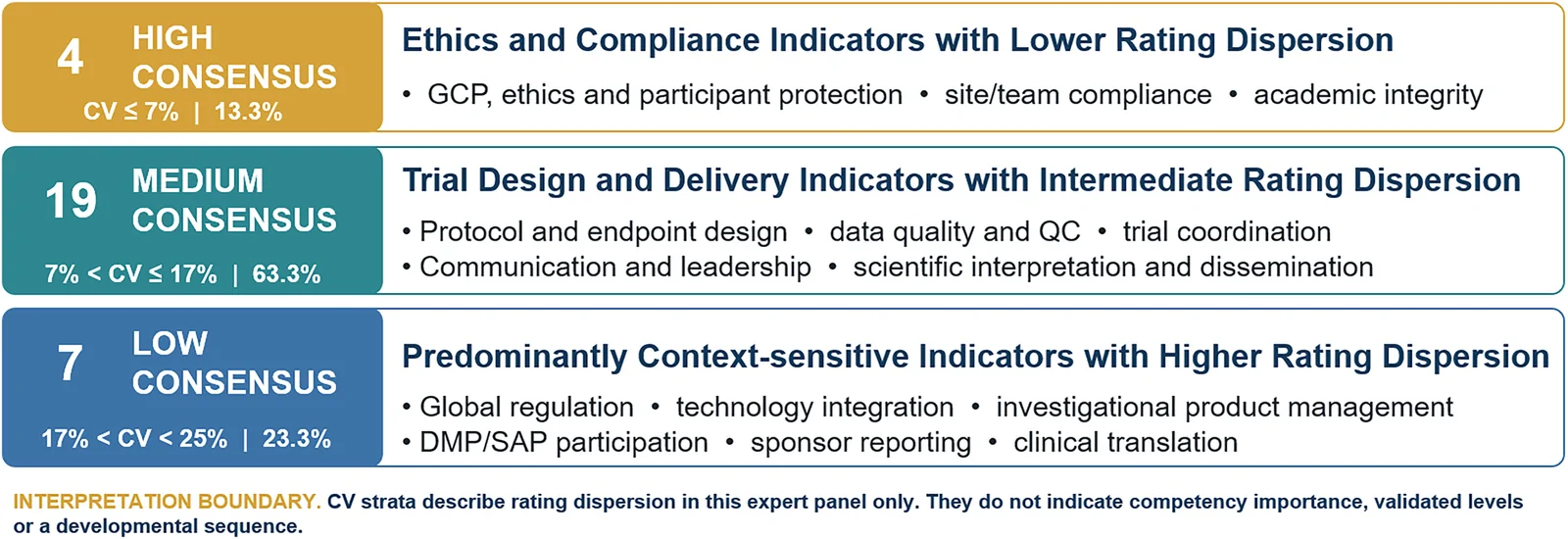
CV-based stratification of 30 PI competency indicators by rating dispersion in a multi-stakeholder Delphi panel.

### 6.2 Exploratory interpretation of indicators with high rating dispersion

Several contextual factors could plausibly contribute to high dispersion, including differences in expert roles, responsibility boundaries, institutional resources and exposure to emerging technologies. Under conventional trial models, PIs have traditionally focused on protocol implementation, whereas the rapid global expansion of China's innovative drug development increasingly requires them to adopt a global perspective. Dispersion in the integration of technological innovations may reflect tensions between established clinical practice models and the adoption of new medical technologies and tools. Uneven digital infrastructure across institutions may confer advantages on early adopters and suggest that future PIs will require stronger data literacy alongside clinical expertise. Refined management of investigational drugs showed the highest dispersion (CV = 23.45%), which may reflect ambiguous boundaries between leadership accountability and operational responsibility. This finding highlights the need to clarify internal procedures and role allocation within institutional management. Dispersion in clinical translation may also reflect the expansion of the PI role and the need to build a collaborative “research–clinical–industry” ecosystem. These interpretations remain exploratory hypotheses that require empirical testing.

### 6.3 Development of a differentiated PI competency framework

Based on the two-round Delphi survey and the current industry context in China, we developed an exploratory conceptual framework for differentiated competency development (Table 3). The considerations presented in Table 3 are hypotheses informed by rating dispersion and indicator content, rather than validated competency requirements or an ordered developmental sequence. PI competency training and assessment could therefore move beyond a one-size-fits-all model toward context-sensitive adaptation. These include the complexity and innovativeness of the trial [e.g., first-in-human (FIH) trials, cell and gene therapies, and trials involving AI-powered imaging analysis]. In these trials, the requirements for competency in integrating new technologies are higher than in conventional small-molecule Phase III trials. Other contextual variables include PI career stage and role definition. Novice PIs could focus on basic competence and independent leadership to ensure safety and compliance. Experienced PIs or academic leaders could aim to develop expertise in other areas and play a strategic leading role. The positioning and resources of the research site are another contextual variable. PIs at top-tier academic medical centers may be expected to engage more in cutting-edge scientific exploration and translational research, while PIs in grassroots research networks may place greater emphasis on high-quality, efficient execution of standardized protocols.

**Table 3:** Exploratory, context-dependent considerations for PI competency development.

Descriptive consensus strata	Descriptive indicator profile	Exploratory competency-development considerations
High-consensus	Four indicators with the lowest rating dispersion, covering academic integrity, regulatory compliance, and research participant protection.	Potential considerations include compliance implementation, risk judgment, team leadership, and awareness of safeguards for data authenticity and academic integrity.
Medium-consensus	Nineteen indicators with intermediate rating dispersion, spanning protocol design, quality control, and internal and external communication and coordination.	Potential considerations include scientific insight, protocol design, management and quality control, communication, team leadership, ethical risk control, and compliance implementation.
Low-consensus (high-divergence)	Seven indicators with the highest rating dispersion, involving global regulatory requirements, emerging technologies, and clinical translation.	Potential considerations include a global perspective, integration of emerging technologies, clinical translation, and coordination across stakeholders. These considerations require prospective validation.

For individual PIs, this inventory may serve as a reference for informal self-reflection and individualized learning plans. For institutions and sponsors, it may provide preliminary insights into potential human-resource management and training applications. Once validated, potential applications could include developing trial-specific competency profiles to inform PI assignment and aligning assessment indicators with trial objectives. They could also include designing modular, elective training systems that address the needs of PIs with different roles and backgrounds. Until then, the inventory should not be used for PI selection or performance appraisal. Industry associations may consider whether the inventory could inform context-specific PI training programs after validation. From a regulatory science perspective, stakeholders could explore whether universal baseline standards should be supplemented with more specific competency expectations for high-risk or technically complex trials. This approach might eventually inform category-specific guidance and support context-sensitive competency development across the industry. These potential applications were not directly tested in the present study and should be treated as hypotheses requiring future validation.

### 6.4 Limitations

This study has several limitations. First, the Delphi method inherently relies on experts' subjective judgment. Although we have ensured quality through rigorous expert selection and authority validation, the practical utility of the competency framework ultimately depends on its prospective validation in real-world clinical trial settings. To this end, we propose that future research adopt a longitudinal, multicenter design, wherein PIs are assessed against the framework at trial initiation, and their subsequent performance and trial outcomes are systematically tracked throughout the trial lifecycle. Key validation metrics should encompass both PI-level behavioral indicators (e.g., team communication, decision-making, problem-solving under pressure) and objective trial quality parameters (e.g., patient enrollment speed, data completeness and accuracy, protocol deviation rates, and timely dissemination of results). Statistical approaches such as multiple regression or mixed-effects models could be used to examine the predictive validity of the competency scores, while adjusting for site-specific and trial-level confounders (e.g., trial phase, therapeutic area, site experience). Additionally, qualitative feedback from PIs and site staff could be collected to evaluate the framework's face validity and practical acceptability. Such prospective validation would not only quantify the framework's impact on PI performance and trial quality, but also identify which competency domains most strongly predict trial success, thereby providing evidence-based guidance for PI selection, training, and mentorship in future clinical trials. Meanwhile, the composition of experts changed in the second round: some first-round experts dropped out, while two additional experts were recruited for role balance. Such inconsistency in the expert panel may introduce selection bias. Newly added experts lacked background information on first-round discussions and consensus evolution, potentially compromising the longitudinal comparability of ratings across Delphi rounds, increasing variability in the statistical indices, and limiting the robustness and reproducibility of the final consensus. Nevertheless, this adjustment represents a methodological trade-off: we prioritized content validity through purposive sampling over longitudinal statistical consistency. While this may affect the comparability of ratings between rounds, it was essential to ensure that the final competency framework possesses the necessary breadth and professional relevance for the Chinese regulatory context.

Second, we acknowledge a key limitation regarding the expert sample. Although we took internal diversity into account during the selection process, the panel size was relatively small and the participants were predominantly recruited from Shanghai, China, which restricts the direct generalizability of our indicator framework to other countries or healthcare systems with different socio-cultural and organizational contexts. Therefore, we emphasize that the current findings should be interpreted with caution, and that cross-cultural validation across broader international settings is essential before the framework can be widely applied. Finally, although the Delphi method yielded a statistically significant consensus (*p* < 0.001), Kendall's coefficient of concordance (*W* = 0.206) indicated only a weak-to-moderate level of agreement among experts. This suggests that, while the consensus is unlikely to be attributable to random chance, there remains considerable divergence in expert opinions regarding the relative importance of specific competencies. This finding is not uncommon in first-round Delphi surveys, particularly when panel members come from heterogeneous professional backgrounds or when the evaluated construct is multifaceted and emergent. The relatively low *W* value implies that the current framework should be viewed as a preliminary prioritization rather than a definitive one. More importantly, this moderate consensus signals that certain competency items may be context-sensitive and therefore require further empirical testing across diverse settings. Thus, beyond additional iterative Delphi rounds to refine expert judgment, future research should examine the practical applicability of these competencies in real-world health-care environments to determine whether the observed divergence reflects genuine contextual differences rather than measurement uncertainty.

## 7 Conclusions

Adopting a systematic and rigorous mixed-methods design with two rounds of the Delphi method, this study developed a core competency framework comprising 30 key items for Principal Investigators (PIs) engaged in innovative drug clinical trials in China. This framework offers an exploratory shift from a static, uniform checklist toward a more differentiated and context-sensitive conceptualization of PI competencies. The proposed differentiated development framework is intended to provide a preliminary theoretical reference and a tool for rethinking the homogenization tendency currently prevalent in PI competency assessment and development.

This framework may ultimately serve as a reference for supporting the development of China's innovative drug clinical trial PI workforce by offering a structured yet flexible lens through which to consider competency development across diverse roles and trial contexts. However, as an exploratory model, it requires further empirical validation before its utility can be confirmed. Future research should prioritize the prospective application and validity testing of this indicator framework in real-world settings, as well as the establishment of a dynamic update mechanism to ensure its continued relevance in a rapidly evolving research and development landscape.

## Data Availability

The raw data supporting the conclusions of this article will be made available by the authors, without undue reservation.
