# Supplementary material for: Beyond GCP: developing a differentiated competency framework for principal investigators in innovative drug clinical trials in China
Source: Front Med (Lausanne). 2026 Sep 23;13:1933721. doi: 10.3389/fmed.2026.1933721 (PMC13647746; doi:10.3389/fmed.2026.1933721)
Supplement: Supplementary file 1 [file Table1.docx]

**Supplementary Table 1.** Descriptive statistics of Round 1 importance ratings for all 64 competency indicators.

| **Primary Domain** | **Code** | **Indicator Item** | **Min** | **Max** | **Mean** | **SD** | **CV** |
| --- | --- | --- | --- | --- | --- | --- | --- |
| **1. Scientific Mindset and Study Design** | 1.1.1 | Analyze the mechanism of action of innovative drugs by integrating pathophysiology and pharmacology to guide intervention design | 2 | 5 | 4.48 | 0.762 | 0.170 |
|  | 1.1.2 | Apply toxicological data to develop safety monitoring parameters based on the development stage of innovative drugs | 2 | 5 | 4.48 | 0.789 | 0.176 |
|  | 1.2.1 | Formulate research hypotheses that address clinical needs based on Chinese epidemiological characteristics of diseases; identify testable scientific questions and translate them into research hypotheses | 3 | 5 | 4.68 | 0.587 | 0.125 |
|  | 1.2.2 | Define efficacy and safety endpoints in compliance with NMPA review requirements | 3 | 5 | 4.82 | 0.438 | 0.091 |
|  | 1.3.1 | Evaluate and optimize study protocols for innovative drugs to ensure scientific rigor and compliance with core principles including randomization, control, and blinding | 2 | 5 | 4.76 | 0.555 | 0.117 |
|  | 1.3.2 | Lead the evaluation and optimization of screening criteria and implementation procedures in multicenter clinical trial protocols to ensure consistency and high quality of research execution across different regions in China | 2 | 5 | 4.58 | 0.702 | 0.153 |
|  | 1.3.3 | Assess the scientific merit and ethical appropriateness of protocols, balancing quality with participant risk | 3 | 5 | 4.70 | 0.580 | 0.123 |
|  | 1.4.1 | Integrate innovative technologies (e.g., novel biomarkers, digital tools) into innovative drug research | 2 | 5 | 3.98 | 0.795 | 0.200 |
|  | 1.4.2 | Promote the standardized application of new technologies such as eCOA and remote monitoring; utilize CTMS and other systems to analyze key performance indicators (KPIs) for data-driven continuous improvement of research implementation and decision-making | 1 | 5 | 3.84 | 0.934 | 0.243 |
|  | 1.5.1 | Critically analyze clinical trial data (assessing the application potential and limitations of research findings) to identify efficacy and safety signals | 3 | 5 | 4.54 | 0.579 | 0.128 |
|  | 1.5.2 | Propose pathways for translating research findings into clinical practice in China | 1 | 5 | 4.24 | 0.822 | 0.194 |
| **2. Ethics and Trial Participant Protection** | 2.1.1 | Explain the exploratory nature of innovative drug research to participants to avoid 'therapeutic misconception' (understanding that clinical trials should follow the principle of 'clinical equipoise,' meaning that the probability of benefit or harm from each treatment arm is approximately equal) | 2 | 5 | 4.58 | 0.731 | 0.160 |
|  | 2.1.2 | Design protocols that clearly distinguish between routine clinical care procedures and research procedures | 2 | 5 | 4.40 | 0.833 | 0.189 |
|  | 2.2.1 | Implement informed consent procedures in accordance with Chinese GCP, ICH GCP, and the Declaration of Helsinki | 1 | 5 | 4.74 | 0.694 | 0.146 |
|  | 2.2.2 | Protect participant data privacy in compliance with the Personal Information Protection Law | 1 | 5 | 4.62 | 0.780 | 0.169 |
|  | 2.2.3 | Understand the principle of 'clinical equipoise' in conducting clinical trials (random assignment of participants to different treatment arms indicates that the medical community is uncertain about the relative effectiveness of the treatments being compared, and that each treatment arm is equally likely to be beneficial or harmful) | 1 | 5 | 4.48 | 0.814 | 0.182 |
|  | 2.3.1 | Identify vulnerable populations (low-income individuals, patients with rare diseases) and develop targeted recruitment strategies | 1 | 5 | 4.16 | 0.976 | 0.235 |
|  | 2.3.2 | Provide additional rights protection measures for special populations or vulnerable groups (elderly, children, pregnant women, persons with disabilities, etc.) to ensure equity | 1 | 5 | 4.28 | 0.904 | 0.211 |
|  | 2.4.1 | Dynamically assess the risk-benefit ratio for participants | 1 | 5 | 4.60 | 0.728 | 0.158 |
|  | 2.4.2 | Master the principles and methods for balancing risks and benefits during participant screening and management | 1 | 5 | 4.38 | 0.878 | 0.200 |
| **3. Innovative Drug R&D and Regulatory Compliance** | 3.1.1 | Master the IND and NDA processes and requirements of the NMPA | 2 | 5 | 4.34 | 0.848 | 0.195 |
|  | 3.1.2 | Understand regulatory requirements from major global regulatory authorities and incorporate them into trial design and execution | 2 | 5 | 4.08 | 0.877 | 0.215 |
|  | 3.2.1 | Understand the full lifecycle process of innovative drugs from preclinical and clinical development through regulatory approval to post-marketing | 2 | 5 | 4.32 | 0.935 | 0.217 |
|  | 3.2.2 | Cooperate with sponsors to complete data collection and documentation preparation at each stage in compliance with Chinese regulatory submission requirements | 2 | 5 | 4.52 | 0.839 | 0.186 |
|  | 3.3.1 | Comply with SAE and SUSAR reporting timelines and procedures required by Chinese regulatory authorities | 2 | 5 | 4.78 | 0.582 | 0.122 |
|  | 3.3.2 | Cooperate in post-marketing adverse event monitoring and pharmacovigilance | 2 | 5 | 4.32 | 0.768 | 0.178 |
|  | 3.4.1 | Coordinate multicenter research in China to ensure consistent protocol implementation | 2 | 5 | 4.62 | 0.697 | 0.151 |
|  | 3.4.2 | Address regional differences to ensure data consistency | 2 | 5 | 4.34 | 0.798 | 0.184 |
| **4. Clinical Research Operation Management** | 4.1.1 | Lead compliance with Chinese GCP and site SOPs | 3 | 5 | 4.82 | 0.438 | 0.091 |
|  | 4.1.2 | Supervise team compliance with operations to ensure all research activities strictly follow the protocol and prevent data fabrication or procedural irregularities | 3 | 5 | 4.82 | 0.482 | 0.100 |
|  | 4.2.1 | Execute procedures for receipt, storage, dispensing, return, and destruction of investigational products for clinical research (including cold chain management) | 2 | 5 | 4.34 | 0.982 | 0.226 |
|  | 4.2.2 | Accurately document drug usage to ensure accountability and inventory accuracy | 2 | 5 | 4.38 | 0.945 | 0.216 |
|  | 4.3.1 | Promptly identify and document AEs and assess their relationship to the investigational product | 2 | 5 | 4.70 | 0.647 | 0.138 |
|  | 4.3.2 | Complete SAE reporting, follow-up, and tracking | 3 | 5 | 4.76 | 0.555 | 0.117 |
|  | 4.4.1 | Cooperate with sponsor and CRO monitoring by providing authentic and complete documentation | 2 | 5 | 4.60 | 0.670 | 0.146 |
|  | 4.4.2 | Lead responses to audits and inspections by NMPA and IRB/EC; based on audit findings, spearhead the development of corrective and preventive actions (CAPA) and optimization of site SOPs | 3 | 5 | 4.74 | 0.487 | 0.103 |
| **5. Research Site and Project Management** | 5.1.1 | Integrate medical resources (wards, laboratories, equipment) to support research implementation | 4 | 5 | 4.80 | 0.404 | 0.084 |
|  | 5.1.2 | Allocate human resources rationally with clear division of labor and responsibilities | 4 | 5 | 4.80 | 0.404 | 0.084 |
|  | 5.2.1 | Lead or deeply participate in negotiation of research contracts and budgets to ensure coverage of full-process costs and compliance with site legal and financial regulations | 3 | 5 | 4.24 | 0.716 | 0.169 |
|  | 5.2.2 | Supervise budget execution to prevent misuse of funds and ensure financial transparency | 3 | 5 | 4.20 | 0.756 | 0.180 |
|  | 5.3.1 | Develop efficient recruitment strategies based on Chinese patients' healthcare-seeking behaviors | 3 | 5 | 4.22 | 0.737 | 0.175 |
|  | 5.3.2 | Design protocols tailored to Chinese patient characteristics to reduce loss to follow-up | 3 | 5 | 4.48 | 0.544 | 0.121 |
|  | 5.4.1 | Develop milestone schedules, dynamically monitor progress, and resolve delays | 3 | 5 | 4.30 | 0.647 | 0.150 |
|  | 5.4.2 | Regularly report research progress to sponsors and IRB/EC | 3 | 5 | 4.14 | 0.783 | 0.189 |
| **6. Data Management and Quality Control** | 6.1.1 | Ensure that source data (medical records, laboratory reports, etc.) are authentic, accurate, and complete | 4 | 5 | 4.86 | 0.351 | 0.072 |
|  | 6.1.2 | Supervise data entry, verification, and correction to minimize data queries | 2 | 5 | 4.18 | 0.896 | 0.214 |
|  | 6.1.3 | Participate in evaluating the Data Management Plan (DMP) and Statistical Analysis Plan (SAP) to ensure their design can scientifically and efficiently achieve research objectives | 2 | 5 | 4.34 | 0.798 | 0.184 |
|  | 6.2.1 | Proficiently use EDC systems for standardized data entry and storage | 1 | 5 | 3.92 | 1.209 | 0.309 |
|  | 6.2.2 | Cooperate in database lock procedures to ensure data are ready for statistical analysis | 1 | 5 | 4.20 | 0.990 | 0.236 |
|  | 6.3.1 | Establish internal site QC processes (regular self-inspections, procedure reviews) | 3 | 5 | 4.62 | 0.530 | 0.115 |
|  | 6.3.2 | Cooperate with external monitoring and audits, rectify quality issues, and continuously improve quality | 3 | 5 | 4.58 | 0.538 | 0.117 |
| **7. Leadership and Professional Literacy** | 7.1.1 | Lead the research team with clear responsibilities to enhance collaboration efficiency | 3 | 5 | 4.78 | 0.465 | 0.097 |
|  | 7.1.2 | Establish team training and development systems to enhance overall team professional capabilities through mentoring and performance management | 3 | 5 | 4.60 | 0.571 | 0.124 |
|  | 7.2.1 | Eliminate academic misconduct (data fabrication, non-compliant informed consent) | 4 | 5 | 4.94 | 0.240 | 0.049 |
|  | 7.2.2 | Proactively declare research-related conflicts of interest in compliance with research integrity requirements | 3 | 5 | 4.80 | 0.452 | 0.094 |
|  | 7.3.1 | Continuously monitor relevant policies and technical advances for innovative drugs in China | 3 | 5 | 4.52 | 0.614 | 0.136 |
|  | 7.3.2 | Integrate new policies and technologies into research practice | 3 | 5 | 4.32 | 0.683 | 0.158 |
| **8. Communication and Collaboration** | 8.1.1 | Communicate efficiently with the team (CRC, CRA, laboratory) to ensure smooth processes | 2 | 5 | 4.66 | 0.626 | 0.134 |
|  | 8.1.2 | Clearly explain research content to participants, answer questions, and enhance compliance | 1 | 5 | 4.54 | 0.762 | 0.168 |
|  | 8.1.3 | Communicate promptly with IRB/EC and NMPA to respond to regulatory requirements | 4 | 5 | 4.68 | 0.471 | 0.101 |
|  | 8.2.1 | Collaboratively resolve research issues (protocol deviations, SAE management) | 4 | 5 | 4.70 | 0.463 | 0.098 |
|  | 8.2.2 | Provide feedback on practical issues to offer decision-making references for sponsors | 4 | 5 | 4.56 | 0.501 | 0.110 |
|  | 8.3.1 | Lead or serve as a major contributor in writing high-quality academic papers and publish research findings in peer-reviewed journals | 2 | 5 | 4.34 | 0.772 | 0.178 |
|  | 8.3.2 | Educate the public on the value of innovative drug research to enhance social awareness | 3 | 5 | 4.28 | 0.671 | 0.157 |
| *Indicators are listed by code within each primary domain. Mean, standard deviation (SD), and coefficient of variation (CV = SD/Mean) are computed from a 5-point Likert scale (1 = extremely unimportant, 5 = extremely important).* | | | | | | | |
