# Supplementary material for: Beyond GCP: developing a differentiated competency framework for principal investigators in innovative drug clinical trials in China
Source: Front Med (Lausanne). 2026 Sep 23;13:1933721. doi: 10.3389/fmed.2026.1933721 (PMC13647746; doi:10.3389/fmed.2026.1933721)
Supplement: Supplementary file 2 [file Table2.docx]

**Supplementary Table 2. Simplified Indicator Set with Mapping to Initial Indicators and Rationale for Item Reduction**

**Note:** *The initial 64 indicators were derived through literature review, semi-structured interviews, and expert consultation. During the two-round Delphi process, indicators with high inter-item correlation and overlapping content were identified for consolidation. The final 30 indicators reflect items achieving consensus (median score ≥ 4 on a 5-point Likert scale) after structural optimization. Items marked with an asterisk (*) were partially merged, meaning only specific elements were absorbed while others informed adjacent indicators.*

**Table 2a. Mapping of 30 Simplified Indicators to Initial 64 Indicators**

| **No.** | **Simplified Indicator (Round 2 Final)** | **Original Item IDs** | **Excluded/Partial Items** | **Rationale for Consolidation or Retention** |
| --- | --- | --- | --- | --- |
| 1 | Integrate pathophysiology, pharmacology, and toxicology to analyze the mechanism of action of innovative drugs, guide intervention design, and formulate safety monitoring indicators. | 1.1.1, 1.1.2 | None | Merged: Both items address biomedical principle application for MoA analysis; combined into a single comprehensive indicator. |
| 2 | Based on epidemiological characteristics of diseases in China, formulate research hypotheses that meet clinical needs; identify testable scientific questions and translate them into research hypotheses. | 1.2.1 | None | Retained as a standalone indicator; no similar items to merge. |
| 3 | Define efficacy/safety endpoints in compliance with NMPA review requirements. | 1.2.2 | None | Retained as a standalone indicator; distinct regulatory focus. |
| 4 | Lead the evaluation and optimization of screening criteria and implementation procedures in multicenter clinical trial protocols to ensure consistency and high-quality research execution across regions in China. | 1.3.2, 3.4.2 | None | Merged: Items from different domains (Protocol Design and Multicenter Coordination) addressed overlapping content on cross-regional consistency; consolidated to avoid redundancy. |
| 5 | Evaluate and optimize trial protocols to ensure scientific rigor and ethical appropriateness, balancing quality with participant risk. | 1.3.1, 1.3.3 | None | Merged: Both items addressed protocol evaluation; 1.3.1 (scientific rigor) and 1.3.3 (ethical appropriateness) were highly overlapping and combined into a unified assessment indicator. |
| 6 | Integrate innovative technologies (e.g., novel biomarkers, digital tools) into innovative drug research to drive data-informed continuous improvement of research implementation and decision-making. | 1.4.1, 1.4.2, 2.3.1 | 2.3.1 (partial) | Merged: 1.4.1 and 1.4.2 both addressed technology adoption in research; combined. 2.3.1 (vulnerable population identification) was partially subsumed as a digital-tool-enabled application (e.g., using CTMS for targeted recruitment). |
| 7 | Critically analyze clinical trial data to identify efficacy and safety signals, assessing the application potential and limitations of research findings. | 1.5.1 | None | Retained as a standalone indicator; no similar items to merge. |
| 8 | Propose pathways for translating research findings into clinical practice in China. | 1.5.2 | None | Retained as a standalone indicator; distinct translational focus. |
| 9 | Explain the exploratory nature of innovative drug research to participants and avoid therapeutic misconception (i.e., understanding the principle of clinical equipoise). | 2.1.1 | None | Retained as a standalone indicator; addresses a unique ethical competency. |
| 10 | Design protocols that clearly distinguish between routine clinical care and research procedures. | 2.1.2 | None | Retained as a standalone indicator; addresses protocol-level ethical distinction. |
| 11 | Comply with Chinese GCP, ICH GCP, the Declaration of Helsinki, and the Personal Information Protection Law; attend to safety event reporting requirements; dynamically assess participant risk–benefit ratio; and provide care for vulnerable populations. | 2.2.1, 2.2.2, 2.2.3, 2.3.2, 2.4.1, 2.4.2, 3.3.1, 8.1.2 | None | Merged: Eight items addressing ethics, participant protection, regulatory compliance, and communication were consolidated. All relate to the overarching competency of ethical and regulatory compliance in participant-facing activities. Expert consensus in Round 2 supported consolidation of highly overlapping participant-protection items. |
| 12 | Understand registration requirements of major global regulatory authorities (including China) and incorporate them into trial design and execution. | 3.1.1, 3.1.2 | None | Merged: 3.1.1 (NMPA-specific) and 3.1.2 (global regulators) were highly related; combined into a single regulatory knowledge indicator. |
| 13 | Cooperate with sponsors to complete data collection and documentation at all stages, meeting Chinese regulatory submission requirements, and understand the full lifecycle of innovative drug R&D. | 3.2.1, 3.2.2 | None | Merged: Both items addressed lifecycle management and sponsor cooperation; combined. |
| 14 | Lead compliance with Chinese GCP and research center SOPs. | 4.1.1 | None | Retained as a standalone indicator; foundational compliance competency. |
| 15 | Supervise team members' compliance to ensure all research activities strictly follow the protocol, preventing data fabrication and procedural irregularities. | 4.1.2, 5.2.1, 5.2.2 | 5.2.1 (partial), 5.2.2 (partial) | Merged: 4.1.2 (protocol compliance supervision) was combined with budget oversight elements from 5.2.1 and 5.2.2, as experts judged that overall operational compliance supervision encompasses financial oversight. |
| 16 | Execute procedures for receipt, storage, dispensing, return, and destruction of investigational products (including cold chain management), with accurate record-keeping to ensure account–inventory consistency. | 4.2.1, 4.2.2 | None | Merged: Both items addressed investigational product management; operational handling (4.2.1) and documentation (4.2.2) were combined. |
| 17 | Regularly report research progress to sponsors and IRB/IEC. | 5.4.2, 5.3.1, 5.4.1 | 5.3.1 (partial) | Merged: Progress reporting (5.4.2), recruitment strategy (5.3.1), and milestone monitoring (5.4.1) were consolidated. Experts considered progress reporting as the umbrella activity encompassing recruitment and timeline tracking. |
| 18 | Ensure source data (medical records, laboratory reports, etc.) are authentic, accurate, and complete. | 6.1.1, 4.3.1 | 4.3.1 (partial) | Merged: Data integrity (6.1.1) and AE identification (4.3.1) were combined, as accurate source data documentation is prerequisite to AE detection. |
| 19 | Participate in evaluating the Data Management Plan (DMP) and Statistical Analysis Plan (SAP) to ensure that research objectives are achieved scientifically and efficiently. | 6.1.3 | None | Retained as a standalone indicator; specialized data governance competency. |
| 20 | Cooperate in database locking to ensure data readiness for statistical analysis. | 6.2.2, 6.1.2, 3.3.2, 3.4.1, 8.1.3 | None | Merged: Five items from different domains converged on data finalization and regulatory cooperation. Experts judged these as operationally interdependent activities in the data-to-analysis pipeline. |
| 21 | Cooperate with external monitoring and auditing; establish internal QC procedures; rectify quality issues for continuous improvement. | 6.3.1, 6.3.2, 4.4.1, 4.4.2 | None | Merged: Internal QC (6.3.1), external audit cooperation (6.3.2), monitoring cooperation (4.4.1), and CAPA development (4.4.2) were combined into a unified quality management indicator. |
| 22 | Establish a team training and development system with clear responsibilities, enhancing overall professional competence through mentoring and performance management. | 7.1.1, 7.1.2, 5.1.1, 5.1.2 | 5.1.1 (partial), 5.1.2 (partial) | Merged: Team leadership (7.1.1, 7.1.2) was combined with resource coordination (5.1.1, 5.1.2), as experts considered human resource allocation integral to team leadership. |
| 23 | Prevent academic misconduct (e.g., data fabrication, non-compliant informed consent). | 7.2.1 | None | Retained as a standalone indicator; core integrity requirement. |
| 24 | Proactively declare research-related conflicts of interest in compliance with research integrity requirements. | 7.2.2 | None | Retained as a standalone indicator; distinct integrity competency. |
| 25 | Continuously monitor policies and technological advances for innovative drugs in China, and integrate new policies and technologies into research practice. | 7.3.1, 7.3.2 | None | Merged: Policy monitoring (7.3.1) and practice integration (7.3.2) represent a continuous improvement cycle; combined. |
| 26 | Communicate efficiently with project teams (CRC, CRA, laboratory) to ensure smooth processes; maintain timely communication with IRB/IEC and NMPA to respond to regulatory requirements. | 8.1.1 | None | Retained as a standalone indicator; consolidated internal and external communication. |
| 27 | Collaboratively resolve research issues (e.g., protocol deviations, SAE management). | 8.2.1, 4.3.2 | 4.3.2 (partial) | Merged: SAE follow-up (4.3.2) was combined with collaborative problem-solving (8.2.1), as SAE management is a key instance of sponsor–PI collaborative resolution. |
| 28 | Provide feedback on practical issues to offer decision-making references for sponsors. | 8.2.2, 5.3.2 | 5.3.2 (partial) | Merged: Practical feedback (8.2.2) and patient retention design (5.3.2) were combined, as retention strategies were considered a form of operational feedback to sponsors. |
| 29 | Lead or serve as a major contributor in writing high-quality academic papers and publishing research findings in peer-reviewed journals. | 8.3.1 | None | Retained as a standalone indicator; core scholarly output competency. |
| 30 | Educate the public on the value of innovative drug research to enhance social awareness. | 8.3.2 | None | Retained as a standalone indicator; distinct public engagement competency. |

Note: In accordance with expert recommendations,Original item 6.2.1, "Proficient Use of EDC Systems for Standardized Data Entry and Storage," has been removed.

**Table 2b. Excluded or Partially Merged Items with Rationale**

*The following original indicators were either fully removed or partially merged into other indicators during the Delphi optimization process.*

| **Original ID** | **Original Indicator Text** | **Reason for Exclusion or Partial Merger** |
| --- | --- | --- |
| 6.2.1 | Proficiently use EDC systems for standardized data entry and storage. | Removed per expert recommendation: EDC operation is a technical skill subsumed under Indicator 20 (database locking cooperation) and Indicator 21 (quality management). Experts considered it too operational for a PI-level competency framework. |
| 5.3.1* | Develop efficient recruitment strategies based on Chinese patients' healthcare-seeking behaviors. | Partially merged into Indicator 17 (progress reporting) and Indicator 28 (feedback to sponsors). Only the strategic planning element was absorbed; the patient-centric design aspect informed Indicator 28. |
| 5.1.1* | Integrate medical resources (wards, laboratories, equipment) to support research implementation. | Partially merged into Indicator 22 (team leadership and resource coordination). The resource allocation aspect was considered integral to team management. |
| 5.1.2* | Allocate human resources rationally with clear division of labor and responsibilities. | Partially merged into Indicator 22 (team leadership). Staffing decisions were judged as a core component of team leadership rather than a standalone competency. |
| 5.2.1* | Lead or participate in research contract and budget negotiation. | Partially merged into Indicator 15 (compliance supervision). Financial oversight was considered part of overall operational compliance. |
| 5.2.2* | Supervise budget execution and ensure financial transparency. | Partially merged into Indicator 15 (compliance supervision). Same rationale as 5.2.1. |
| 2.3.1* | Identify vulnerable populations and develop targeted recruitment strategies. | Partially merged into Indicator 6 (technology integration). The identification and targeted recruitment aspect was subsumed under data-driven recruitment tools. |
| 4.3.1* | Promptly identify and document AEs and assess their relationship to investigational product. | Partially merged into Indicator 18 (source data integrity). AE documentation was considered part of ensuring complete and accurate source data. |
| 4.3.2* | Complete SAE reporting, follow-up, and tracking. | Partially merged into Indicator 11 (attend to safety event reporting requirements) and Indicator 27 (collaborative problem-solving). SAE reporting is a component of overall safety event reporting, and SAE management was considered a key instance of PI–sponsor collaborative resolution. |
