# Supplementary material for: Beyond GCP: developing a differentiated competency framework for principal investigators in innovative drug clinical trials in China
Source: Front Med (Lausanne). 2026 Sep 23;13:1933721. doi: 10.3389/fmed.2026.1933721 (PMC13647746; doi:10.3389/fmed.2026.1933721)
Supplement: Supplementary file 3 [file Table3.docx]

**Supplementary Table 3. Consensus ratings and hierarchical cluster membership of the 30 Optimized competency indicators retained after the two-round Delphi process**

| **No.** | **Optimized Indicator** | **Min** | **Max** | **Mean** | **SD** | **CV** | **Tiers based on CV** | **Cluster** |
| --- | --- | --- | --- | --- | --- | --- | --- | --- |
| 1 | Integrate pathophysiology, pharmacology, and toxicology to analyze the mechanism of action of innovative drugs, guide intervention design, and formulate safety monitoring indicators. | 3 | 5 | 4.58 | 0.720 | 15.72% | Medium | 1 |
| 2 | Based on epidemiological characteristics of diseases in China, formulate research hypotheses that meet clinical needs; identify testable scientific questions and translate them into research hypotheses. | 2 | 5 | 4.55 | 0.723 | 15.89% | Medium | 1 |
| 3 | Define efficacy/safety endpoints in compliance with NMPA review requirements. | 3 | 5 | 4.81 | 0.543 | 11.29% | Medium | 2 |
| 4 | Lead the evaluation and optimization of screening criteria and implementation procedures in multicenter clinical trial protocols to ensure consistency and high-quality research execution across regions in China. | 4 | 5 | 4.77 | 0.425 | 8.91% | Medium | 2 |
| 5 | Evaluate and optimize trial protocols to ensure scientific rigor and ethical appropriateness, balancing quality with participant risk. | 4 | 5 | 4.84 | 0.374 | 7.73% | Medium | 2 |
| 6 | Integrate innovative technologies (e.g., novel biomarkers, digital tools) into innovative drug research to drive data-informed continuous improvement of research implementation and decision-making. | 3 | 5 | 4.13 | 0.718 | 17.38% | Low | 2 |
| 7 | Critically analyze clinical trial data to identify efficacy and safety signals, assessing the application potential and limitations of research findings. | 3 | 5 | 4.48 | 0.677 | 15.11% | Medium | 2 |
| 8 | Propose pathways for translating research findings into clinical practice in China. | 3 | 5 | 4.39 | 0.761 | 17.33% | Low | 2 |
| 9 | Explain the exploratory nature of innovative drug research to participants and avoid therapeutic misconception (i.e., understanding the principle of clinical equipoise). | 3 | 5 | 4.61 | 0.615 | 13.34% | Medium | 2 |
| 10 | Design protocols that clearly distinguish between routine clinical care and research procedures. | 3 | 5 | 4.55 | 0.568 | 12.48% | Medium | 3 |
| 11 | Comply with Chinese GCP, ICH GCP, the Declaration of Helsinki, and the Personal Information Protection Law; attend to safety event reporting requirements; dynamically assess participant risk–benefit ratio; and provide care for vulnerable populations. | 4 | 5 | 4.90 | 0.301 | 6.14% | High | 1 |
| 12 | Understand registration requirements of major global regulatory authorities (including China) and incorporate them into trial design and execution. | 2 | 5 | 4.23 | 0.884 | 20.90% | Low | 2 |
| 13 | Cooperate with sponsors to complete data collection and documentation at all stages, meeting Chinese regulatory submission requirements, and understand the full lifecycle of innovative drug R&D. | 2 | 5 | 4.42 | 0.848 | 19.19% | Low | 2 |
| 14 | Lead compliance with Chinese GCP and research center SOPs. | 4 | 5 | 4.87 | 0.341 | 7.00% | High | 2 |
| 15 | Supervise team members' compliance to ensure all research activities strictly follow the protocol, preventing data fabrication and procedural irregularities. | 4 | 5 | 4.90 | 0.301 | 6.14% | High | 2 |
| 16 | Execute procedures for receipt, storage, dispensing, return, and destruction of investigational products (including cold chain management), with accurate record-keeping to ensure account–inventory consistency. | 2 | 5 | 4.26 | 0.999 | 23.45% | Low | 2 |
| 17 | Regularly report research progress to sponsors and IRB/IEC. | 2 | 5 | 4.39 | 0.761 | 17.33% | Low | 2 |
| 18 | Ensure source data (medical records, laboratory reports, etc.) are authentic, accurate, and complete. | 2 | 5 | 4.84 | 0.583 | 12.05% | Medium | 2 |
| 19 | Participate in evaluating the Data Management Plan (DMP) and Statistical Analysis Plan (SAP) to ensure that research objectives are achieved scientifically and efficiently. | 3 | 5 | 4.19 | 0.749 | 17.88% | Low | 1 |
| 20 | Cooperate in database locking to ensure data readiness for statistical analysis. | 2 | 5 | 4.58 | 0.720 | 15.72% | Medium | 2 |
| 21 | Cooperate with external monitoring and auditing; establish internal QC procedures; rectify quality issues for continuous improvement. | 3 | 5 | 4.68 | 0.541 | 11.56% | Medium | 2 |
| 22 | Establish a team training and development system with clear responsibilities, enhancing overall professional competence through mentoring and performance management. | 4 | 5 | 4.71 | 0.461 | 9.79% | Medium | 2 |
| 23 | Prevent academic misconduct (e.g., data fabrication, non-compliant informed consent). | 4 | 5 | 4.90 | 0.301 | 6.14% | High | 2 |
| 24 | Proactively declare research-related conflicts of interest in compliance with research integrity requirements. | 4 | 5 | 4.74 | 0.445 | 9.39% | Medium | 2 |
| 25 | Continuously monitor policies and technological advances for innovative drugs in China, and integrate new policies and technologies into research practice. | 3 | 5 | 4.42 | 0.620 | 14.03% | Medium | 2 |
| 26 | Communicate efficiently with project teams (CRC, CRA, laboratory) to ensure smooth processes; maintain timely communication with IRB/IEC and NMPA to respond to regulatory requirements. | 3 | 5 | 4.81 | 0.477 | 9.92% | Medium | 2 |
| 27 | Collaboratively resolve research issues (e.g., protocol deviations, SAE management). | 4 | 5 | 4.81 | 0.402 | 8.36% | Medium | 2 |
| 28 | Provide feedback on practical issues to offer decision-making references for sponsors. | 3 | 5 | 4.45 | 0.568 | 12.76% | Medium | 2 |
| 29 | Lead or serve as a major contributor in writing high-quality academic papers and publishing research findings in peer-reviewed journals. | 3 | 5 | 4.39 | 0.715 | 16.29% | Medium | 2 |
| 30 | Educate the public on the value of innovative drug research to enhance social awareness. | 3 | 5 | 4.32 | 0.653 | 15.12% | Medium | 2 |
| **Note:** *Mean, standard deviation (SD), and coefficient of variation (CV = SD/Mean) are computed from Round 2 importance ratings on a 5-point Likert scale (1 = extremely unimportant, 5 = extremely important).* *Across the 30 optimized indicators, Round 2 mean importance ratings ranged from 4.13 to 4.90 (mean = 4.58), and CV values ranged from 0.061 to 0.235. Indicators are classified into high-, medium-, and low-consensus tiers on the basis of CV. The final column reports cluster membership from a hierarchical cluster analysis of the Round 2 rating profiles. Four indicators (13.3%) met the high-consensus criterion, 19 (63.3%) were classified as medium consensus, and seven (23.3%) as low consensus. Hierarchical cluster analysis assigned the 30 indicators to three clusters (n = 4, 25, and 1, respectively).* | | | | | | | | |
