# Supplementary material for: Beyond GCP: developing a differentiated competency framework for principal investigators in innovative drug clinical trials in China
Source: Front Med (Lausanne). 2026 Sep 23;13:1933721. doi: 10.3389/fmed.2026.1933721 (PMC13647746; doi:10.3389/fmed.2026.1933721)
Supplement: Supplementary file 4 [file Table4.docx]

**Supplementary Table 4. Structured comparison of existing clinical-research competency frameworks and the present PI competency inventory**

| **Framework** | **Intended users** | **Competency domains** | **Number of competency items** | **Assessment purpose** | **Applicability to the Chinese PI context** | **Practical burden / implementation considerations** |
| --- | --- | --- | --- | --- | --- | --- |
| **JTF Core Competency Framework [13, 21]** | Clinical research professionals across sites, sponsors, CROs, and related roles, including investigators | 8 domains: scientific concepts and research design; ethics and participant safety; investigational products and regulation; clinical study operations; study and site management; data management and informatics; leadership and professionalism; communication and teamwork | 47-49 core competency statements depending on framework version; competencies are levelled as fundamental, skilled, and advanced | Harmonised workforce standards; curricula and training; role descriptions; performance assessment; accreditation and career development | Available internationally and translated into Chinese, but not specifically developed for Chinese PI responsibilities or innovative-drug trial settings | Comprehensive and multi-role. Application requires selection by role, mapping across proficiency levels, and local adaptation. Published sources do not quantify completion time. |
| **TDR Global Competency Framework for Clinical Research [16]** | The clinical research team as a whole, derived from 11 broad research roles and intended for diverse global settings | 5 areas: ethics, quality and risk management; study and site management; research operations; scientific thinking; professional skills | 50 competencies | Capacity strengthening; recognition of skills; gap analysis; role-specific development; career progression; team and individual assessment | Designed for global adaptability, including resource-variable settings, but not PI-specific and not tailored to China's regulatory and institutional arrangements | Flexible wheel, dictionary, grading system, radar tool, and role-specific frameworks. PI use requires selecting and adapting relevant competencies from a whole-team framework. |
| **ACRP Functional Competency Guidelines for Principal and Sub-Investigators [19]** | Principal investigators and sub-investigators; also used by sites and other stakeholders responsible for investigator development | Task-based expectations covering PI oversight and delegation, protocol and GCP compliance, ethics and participant protection, investigational-product management, site and team management, data, and communication | A fixed item count is not reported in the publicly available overview | Training and mentoring of new investigators; competency-based job descriptions; SOP refinement; oversight and delegation expectations | Directly PI-specific and operationally oriented, but not explicitly developed or validated for the Chinese regulatory and healthcare-system context | Actionable guidance rather than a publicly specified concise scoring inventory. Local regulatory mapping and assessment rules are still required. |
| **Present 30-item PI competency inventory** | PIs conducting innovative drug clinical trials in China; regulatory authorities, clinical trial institutions and sponsors | Three dimensions were identified based on consensus levels: high‑consensus domain (trial compliance, research integrity, and protection of trial participants); moderate‑consensus domain (protocol design, quality assurance, internal and external communication); and low‑consensus domain (integration of new technologies, global vision, among others). | 30 indicators refined from an initial pool of 64 | Exploratory development of streamlined PI competency indicators, provide a reference for regulatory authorities, clinical trial institutions, PIs, and sponsor, lay a foundation for subsequent validation. | Explicitly developed for China's innovative-drug trial context and aligned with four stakeholder perspectives | More concise for routine use, but consensus-based and exploratory. Prospective validity, scoring rules, feasibility, and practical utility remain to be established. |

*Note: JTF counts have changed across versions. Version 2.0 contained 47 core competencies, whereas Version 3.1 was reported as containing 49. Implementation considerations are analytical comparisons based on framework scope and public implementation materials; they are not measured completion-time estimates.*

[13] Sonstein SA, Silva H, Jones CT, Bierer BE. Education and training of clinical research professionals and the evolution of the Joint Task Force for Clinical Trial Competency. Front Pharmacol. 2024;15:1291675. doi:10.3389/fphar.2024.1291675.

[16] Julé A, Furtado T, Boggs L, et al. Developing a globally applicable evidence-informed competency framework to support capacity strengthening in clinical research. BMJ Glob Health. 2017;2:e000229. doi:10.1136/bmjgh-2016-000229.

[19] Association of Clinical Research Professionals. ACRP Functional Competency Guidelines for Principal and Sub-Investigators. 2021.

[21] Joint Task Force for Clinical Trial Competency. JTF Core Competency Framework.
